# YOLOv5-LiNet: A lightweight network for fruits instance segmentation

**DOI:** 10.1371/journal.pone.0282297

**Published:** 2023-03-02

**Authors:** Olarewaju Mubashiru Lawal

**Affiliations:** Sanjiang Institute of Artificial Intelligence & Robotics, Yibin University, Sichuan, China; University of Wisconsin-Eau Claire, UNITED STATES

## Abstract

To meet the goals of computer vision-based understanding of images adopted in agriculture for improved fruit production, it is expected of a recognition model to be robust against complex and changeable environment, fast, accurate and lightweight for a low power computing platform deployment. For this reason, a lightweight YOLOv5-LiNet model for fruit instance segmentation to strengthen fruit detection was proposed based on the modified YOLOv5n. The model included Stem, Shuffle_Block, ResNet and SPPF as backbone network, PANet as neck network, and EIoU loss function to enhance detection performance. YOLOv5-LiNet was compared to YOLOv5n, YOLOv5-GhostNet, YOLOv5-MobileNetv3, YOLOv5-LiNetBiFPN, YOLOv5-LiNetC, YOLOv5-LiNet, YOLOv5-LiNetFPN, YOLOv5-Efficientlite, YOLOv4-tiny and YOLOv5-ShuffleNetv2 lightweight model including Mask-RCNN. The obtained results show that YOLOv5-LiNet having the box accuracy of 0.893, instance segmentation accuracy of 0.885, weight size of 3.0 MB and real-time detection of 2.6 ms combined together outperformed other lightweight models. Therefore, the YOLOv5-LiNet model is robust, accurate, fast, applicable to low power computing devices and extendable to other agricultural products for instance segmentation.

## 1. Introduction

The agricultural sector is one major driver of any economy that has to cope with the increasing food consumption as a result of an increase in population. Fruit is an important agricultural product that is not exempted of these consumer demands. The annual worldwide production of some fruits reported in the year 2020 is estimated over 841 million metric tons according to Shahbandeh [1]. An automatic recognition system that comprised of computer vision and personal computer (PC) was introduced to agriculture for the improvement of fruits production. For example, the visual detection techniques used in the horticulture research field to understand fruit-related phenotypic traits, such as number, size, shape and color has replaced the traditional way for monitoring fruit phenotypes, which is destructive and time-consuming. The computer vision captures fruits images while the PC with an integrated deep learning recognition model is used to recognize and locate the target fruits in an image. Using harvesting robot as a case study, the obtained detection results through the recognition model serves as a guide for a manipulator to pick or harvest the fruits. However, the recognition model of either fruits detection or instance segmentation is faced with some impeding factors of complex and changeable environment. To meet the goals of vision-based understanding of images, it is expected of a robust recognition model to be fast, accurate and lightweight for a low power computing platform deployment. This paper proposes a lightweight YOLOv5-LiNet model for fruits instance segmentation based on YOLOv5n to address the shortcomings. The contributions are as follows:

1. A robust cucurbit fruits image dataset with bounding polygon annotation was produced for comparative experiments towards instance segmentation accomplishment.
2. Replace the first layer of the backbone with Stem network to effectively improve the feature expression capability without adding too much computational cost.
3. Incorporate the ShuffleNetv2 network to mix the extracted features, reduce the computational cost and parameters while maintaining accuracy with an improved speedup.
4. The introduction of ResNet network is to improves the efficiency of deep neural networks while minimizing degradation.
5. The application of EIoU loss function is to bring significant and consistent improvements to detection performance.

The rest of this paper is as follows: Section 2 focuses on the work related to fruit detection and instance segmentation. Section 3 describes the details of dataset, proposed model and experiment. Section 4 provides the compared results and discussion of models, and Section 5 concludes.

## 2. Related work

The computer-based recognition model produced by deep learning with convolutional neural networks (CNN) has been able to attain state-of-the-art accuracy, sometimes exceeding human-level with well-known performance in image classification [2], object detection [3, 4] and instance segmentation [5]. Object detection can simultaneously classify and localize each target using a bounding box, and is capable enough to deal with multi-class scenario. With this, deep learning with computer vision has significantly improved the production of fruits through fruit detection for yield prediction, yield estimation, harvesting robot platform, fruit-quality detection, ripeness identification etc. according to Koirala et al. [6], Koirala et al. [7] and Lawal [8, 9]. Notwithstanding, fruit detection scenarios have rectangular bounding boxes and cannot accurately estimate area or perimeter of target from image, in this case, instance segmentation was introduced to consolidates object detection. The instance segmentation technique is more granular with every pixel of given object characterization, that determines the target shape.

Mask-RCNN proposed by He et al. [5] is a deep learning architecture of two-stage detector commonly used for instance segmentation. RiceNet based on improved Mask-RCNN was introduced by Shang et al. [10] for adhesive rice grains segmentation. The RiceNet with few structural parameters recorded an accuracy and recall rate of 89.5% and 92.6% respectively but the target category is single and was not tested on fruits. Liu et al. [11] reported an accuracy of 89.47% and detection time of 346.1 milliseconds (ms) on improved Mask-RCNN for cucumber instance segmentation. However, the speed is slower and robustness is questionable due to one specified category. Yu et al. [12] demonstrated an improved universality and robustness using Mask-RCNN to detect ripe and unripe strawberries but also with slower detection speed. The proposed convolutional encoder–decoder network by Ilyas et al. [13] used adaptive receptive field, channel selection module and bottleneck module to realize accurate recognition of strawberry fruit maturity and diseased fruit but the model could not segment a single target. The optimized Mask-RCNN conducted by Jia et al. [14] on persimmons instance segmentation achieved mean average precision (mAP) and mean average recall (mAR) of 76.3 and 81.1%, respectively. The proposed model is said to be a lightweight network using MobileNetv3 [15] as backbone, but was not tested for detection speed to ascertain the performance, and accuracy requires further improvement. A significant improvement of Mask-RCNN for segmentation of fruit and vegetables was reported by Hameed et al. [16]. However, the experiment may have limitation in cases where the supermarket environment is different from natural environment. Interestingly, most of the research conducted on fruits instance segmentation applied Mask-RCNN, whose model weight tends to be large with slower detection speed. Little or no literature was reported using a single-stage detector for fruits instance segmentation in recent. According to Koirala et al. [7], the speed of a single-stage is faster than two-stage detector, and a fast detector is attributed to the lightweight size of model with reference to Lawal [8].

A single-stage detector DaSNet-v2 of lightweight was experimented by Kang and Chen [17]. It combined fruit detection and instance segmentation, and semantic segmentation of branches into a single network architecture to realize an accurate recognition of fruits in complex orchard environment. At the same demonstrated a weight size of 8.1MB with inference time of 55 ms but still need further improvement. The use of bounding polygons for instance segmentation was first developed by Hurtik et al. [18] named Poly-YOLO. It generates a number of flexible points for the bounding polygons of an object that allows network to be trained for general objects shapes and optimizes the conventional hyper-column to attain a lower loss with the modification of feature maps fusion. As a result of this recent trend, Mirror-YOLO was proposed by Li et al. [19] for the instance segmentation and detection of mirrors. Mirror-YOLO achieved a better performance compared to other existing mirrors detection technique. The motivation behind this work led to the recent introduction of YOLOv5 segmentation by Jocher et al. [20]. YOLOv5 have shown to be outstanding, particularly in lightweight size and speed using the its detection platform, yet to be investigated for fruits instance segmentation. Therefore, it is necessary to develop and evaluate a lightweight fruits instance segmentation model using YOLOv5 framework with special attention to accuracy and speed. Meanwhile, the actualization of lightweight network depends on the application of comparative simpler network structure such as MobileNet (MobileNetv1 [21]; MobileNetv2 [22]; MobileNetv3 [15]), SqueezeNet (SqueezeNet [23]; SqueezeNext [24]), ShuffleNet (ShuffleNetv1 [25]; ShuffleNetv2 [26]), and YOLO-tiny (YOLOv3-tiny [27]; YOLOv4-tiny [28]; YOLOv5n [20]) and so on. For the computer vision system aiming at accurate location and segmentation has a vital role for various agricultural applications [29, 30].

## 3. Methodology

### 3.1 Dataset

With special consideration to reflection, shadows, low light cloudy and high light of environmental factors, the images of cucurbit fruit dataset used in this paper were obtained from wanghaizhuang greenhouses, Jinzhong, Shanxi, China, which are publicly open to society. Cucurbit is a family of fruit plant that shared similarity in ground development and have high genetic diversity in shape, color and size, making the intelligent perception and acquisition of their information most difficult for the fruit instance segmentation. Nevertheless, they are a good source of many nutrients to the human body. For this work, the classes of cucurbit images captured are bitter-melon, cucumber, muskmelon and melon-boyang. These images were taken using a regular 3968×2976 pixels digital camera in the morning, midday and afternoon. A 665 of bitter-melon, 664 of cucumber, 404 of muskmelon and 736 of melon-boyang, making a total of 2469 images were captured, including complex conditions: leaf occlusion, superimposed fruit, dense target, branch occlusion, backlight, front light and other fruit scenes. The collected images were stored in JPG and randomly divided into 80% train-set, 15% valid-set, and 5% test-set. Later, all the ground truth bounding polygons of each target in an image was manually hand labeled using Labelme [31] annotation tool. The purported shape of the target was drawn neglecting the image complex and changeable condition, and annotation files saved in coco format. The obtained coco annotation was converted into poly-YOLO format. The format first takes object class number followed by xy to x_n_ y_n_ for instance segmentation, where xy is the coordinate for n polygon point of mask.

### 3.2 YOLOv5-LiNet

**Fig 1:**
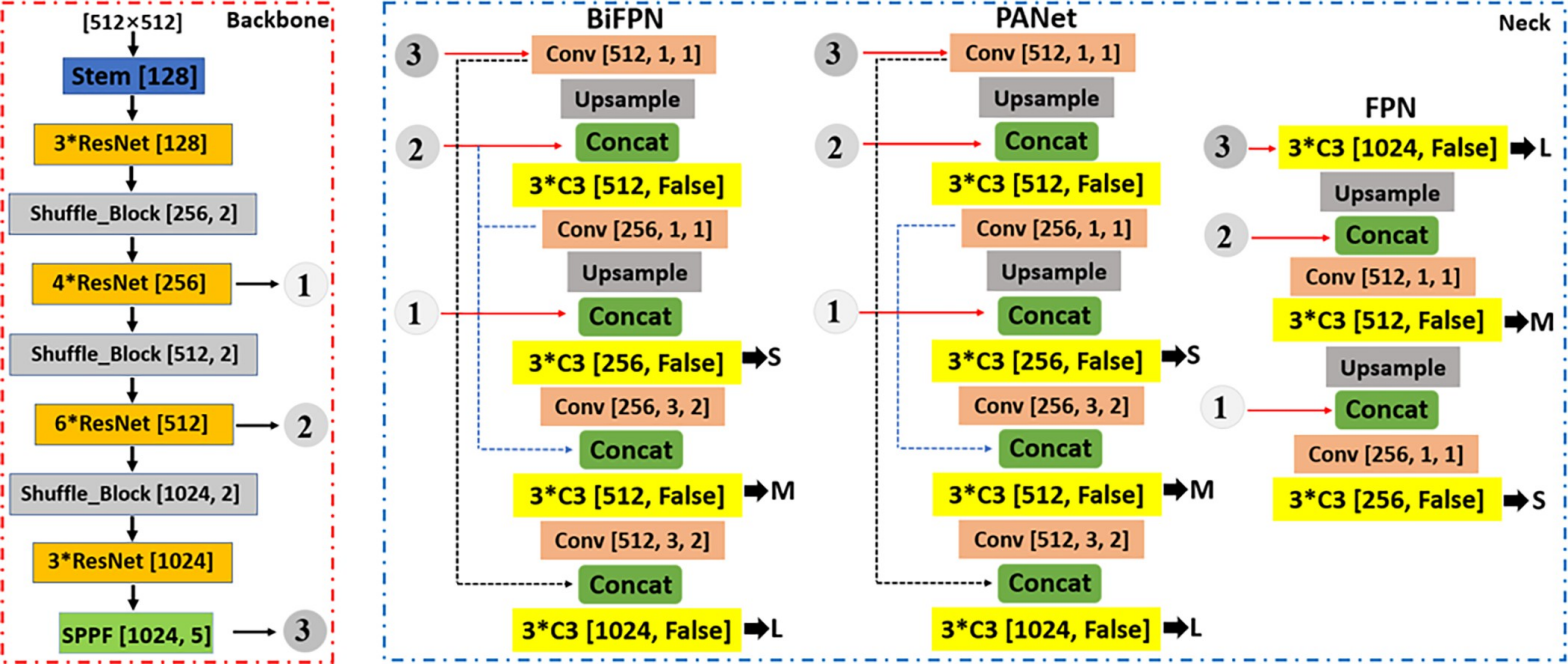
Network of YOLOv5-LiNet. LiNet backbone including neck of BiFPN, PANet and FPN.

**Fig 2:**
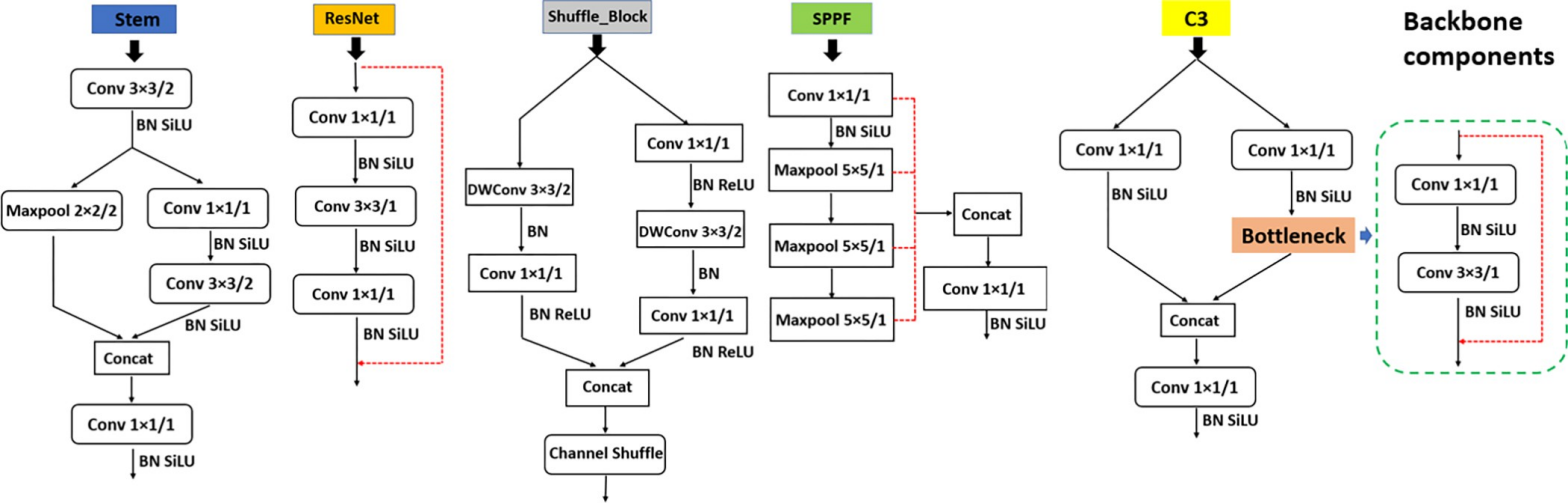
Backbone component of YOLOv5-LiNet. Stem, ResNet, Shuffle_Block, SPPF and C3 incorporated into neck network.

The lightweight YOLOv5-LiNet is designed based on the original YOLOv5n architecture for fruits instance segmentation. It combines the backbone, neck and head network of 0.33 depth and 0.25 width multiple. Generally, backbone network aggregates and forms image features at different granularities. The LiNet backbone of YOLOv5-LiNet shown in Fig 1 comprised of Stem [32], ResNet [33], Shuffle_Block [26] and SPPF [20]. The Stem structure in Fig 2 which is used to replace the original Focus/Conv layer in YOLOv5 consists of Conv layer, Maxpool layer, batch normalization (BN) layer, and a SiLU [34] activation function. Stem as the first spatial down-sampling on the input image increases the generalization of network capability and reduces the computation complexity without performance degradation. The ResNet network in Fig 2 placed after Stem and Shuffle_Block is used to solve drops off from saturated accuracy for deeper neural network. It consists of Conv layer 1×1, 3×3 and 1×1 stacked together according to output identity mappings through the shortcut connection. Conv layer 1×1 is responsible for reducing complexity and parameters. Meanwhile, each of the Conv layer is activated with SiLU to improves deep neural network performance after BN layer. Shuffle_Block shown in Fig 2 utilizes two operations of pointwise group convolution and channel shuffle for spatial down-sampling to reduce computation cost while maintaining accuracy. Pointwise group convolution used a single convolutional filter per each input channel, while the channel shuffle enables information communication between the two concatenated network branches to improved performance. The extracted feature maps after ResNet network were passed to the SPPF in Fig 2. The SPPF is an improvement on spatial pyramid pooling (SPP) [35], which consists of Conv layer with BN layer and SiLU activation, and Maxpool layer. It is a feature enhancement network that extracts the major information of feature map and performs stitching to reduce loss of target detection. SPPF is faster with less giga floating-point operations per second (GFLOPs) compared to SPP according to Jocher et al. [20]. The stated components of YOLOv5-LiNet backbone were chosen to foster less parameters and GFLOPs toward an improve detection accuracy and speed with smaller weight size of model.

Neck network is an important aspect of fruits instance segmentation model that is used to get feature pyramids, and for multi-scale feature extraction in the target detection process. Fig 1 provides different types of neck network used in this paper for ablation study. The path aggregation network (PANet) [36] was fed to YOLOv5-LiNet as neck in order to promote and maintain a balance between accuracy and speed. PANet enables a well-generalized model on object scaling with an incorporation of C3 network shown in Fig 2 and enhances multi-scale fusion. This is to improve detection accuracy. The bottleneck of stack two Conv layers 1×1 and 3×3 with skip connections were embedded into the C3 network after second branch Conv layer 1×1 and later concatenated with the first branch Conv layer 1×1, followed by Conv layer 1×1 to improve detection performance. Meanwhile, each Conv layer is associated with BN layer and SiLU activation. Similarly, the map features from the backbone network were forwarded to neck networks in Fig 1 for convolution and up-sample to produce double image dimensions for concatenation. The concatenated information passes to C3 network for output detection. This process is repeated till small, medium and large level are produce. The head network is the final output of detection. It output both fruit detection and instance segmentation through small, medium and large scale that consumes features from the neck. It adopts bounding polygons (anchors) on mapped features with the probability of the fruit target class, score and position, and non-maximum suppression (NMS) to select the appropriate fruit target and remove redundant information. To measure the quality of model prediction and show the gap between predicted and actual value, Efficient intersection-over-union (EIoU) loss function (see more details by Zhang et al., 2022) [37] was applied to lightweight YOLOv5-LiNet against the commonly used CIoU [38]. EIoU directly measures the overlap area, central point and side length of targets, and anchor for convergence speed and localization accuracy.

### 3.3 Experiment

This experiment deploys python 3.19.13 and torch-1.11.0+cu113 deep learning framework for model training and testing on a computer with an Intel Core i7-12700 CPU @ 64-bit 4.90 GHz, 32 GB RAM, NVIDIA GeForce RTX 3060 12045MiB GPU graphics card and ubuntu22.04LTS operating system. Table 1 provides the details of all the trained models. Using the general procedures for network training on YOLOv5 platform, the proposed lightweight YOLOv5-LiNet including other YOLO related models takes an input of 512×512 pixels, 16 batch size, 0.937 momentum, 0.0005 weight decay, 0.2 IoU, 0.015 hue, 0.7 saturation, 0.4 lightness, 1.0 mosaic and 300 epochs training, while Mask-RCNN received an input of 512×512 pixels with default parameters on MMdetection platform. Random initialization technique was used to initialize the weights for training all the models from scratch.

**Table 1:** List of trained models.

Models	Backbone	Neck	Loss
Mask-RCNN [5]	ResNet50	FPN	Cross-entropy
YOLOv4-tiny [28]	CSPDarkNet	PANet	CIoU
YOLOv5-Efficientlite [39]	EfficientNet	PANet	CIoU
YOLOv5-MobileNetv3 [39]	MobileNetv3	PANet	CIoU
YOLOv5-GhostNet [20]	C3-GhostNet	PANet	CIoU
YOLOv5-ShuffleNetv2 [39]	ShuffleNetv2	PANet	CIoU
YOLOv5n [20]	C3-CSPNet	PANet	CIoU
**Ablation study**			
YOLOv5-LiNetFPN	LiNet	FPN	EIoU
YOLOv5-LiNetBiFPN	LiNet	BiFPN	EIoU
YOLOv5-LiNetC	LiNet	PANet	CIoU
YOLOv5-LiNet	LiNet	PANet	EIoU

### 3.4 Evaluation

This paper used Precision, Recall, F_1_-score and mean Average Precision (mAP) as the evaluation metrics, set at 0.5 IoU threshold. A predicted bounding polygon is correct (true positive) if it overlaps more than the IoU threshold with a labeled bounding polygon, else the predicted bounding polygon is considered false positive. Likewise, it is considered false negative when the labeled bounding polygon has an IoU with a predicted bounding polygon lower than the threshold value. Precision is the ratio of correctly detected fruit to the total number of detected fruits. Recall is the ratio of correctly detected fruit to the total number of fruits in the dataset. F_1_-score is the trade-off between Precision and Recall to show the model performance and mAP is the overall performance under different confidence thresholds [8]. The metrics can be defined as below:

$$\mathrm{Precision}=\frac{\mathrm{TP}}{\mathrm{TP}+\mathrm{FP}}$$ (1)

$$\mathrm{Recall}=\frac{\mathrm{TP}}{\mathrm{TP}+\mathrm{FN}}$$ (2)

$$F_{1}=\frac{2\times \mathrm{Recall}\times \mathrm{Precision}}{\mathrm{Recall}+\mathrm{Precision}}$$ (3)

mAP=AP(Precision,Recall) (4)

TP is True Positive (correct detections), FN is False Negative (missed detections), FP is False Positive (incorrect detections) and AP (•) is the area calculation function under Precision and Recall curves.

## 4. Results and discussion

**Fig 3:**
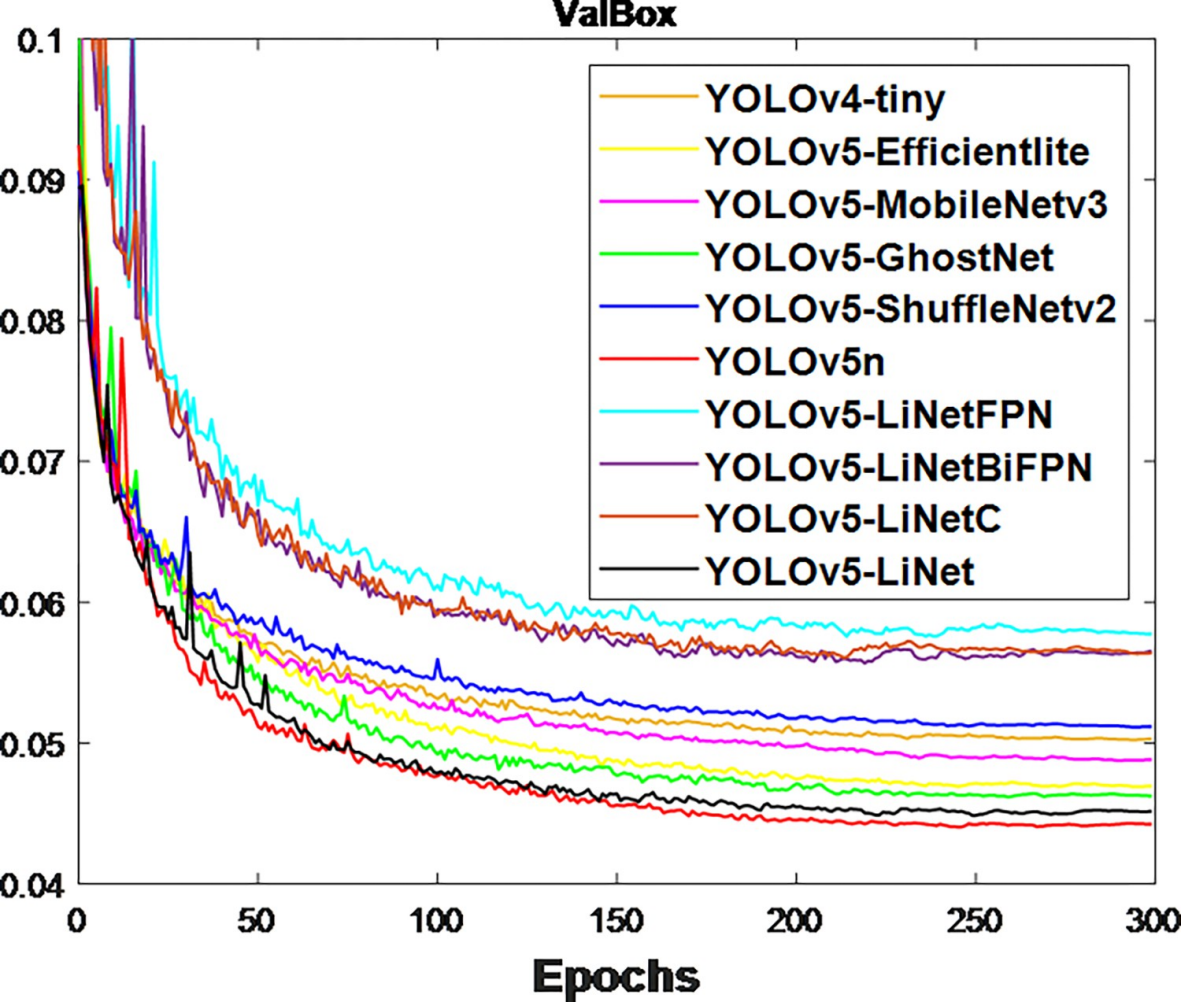
Output for box validation loss.

**Fig 4:**
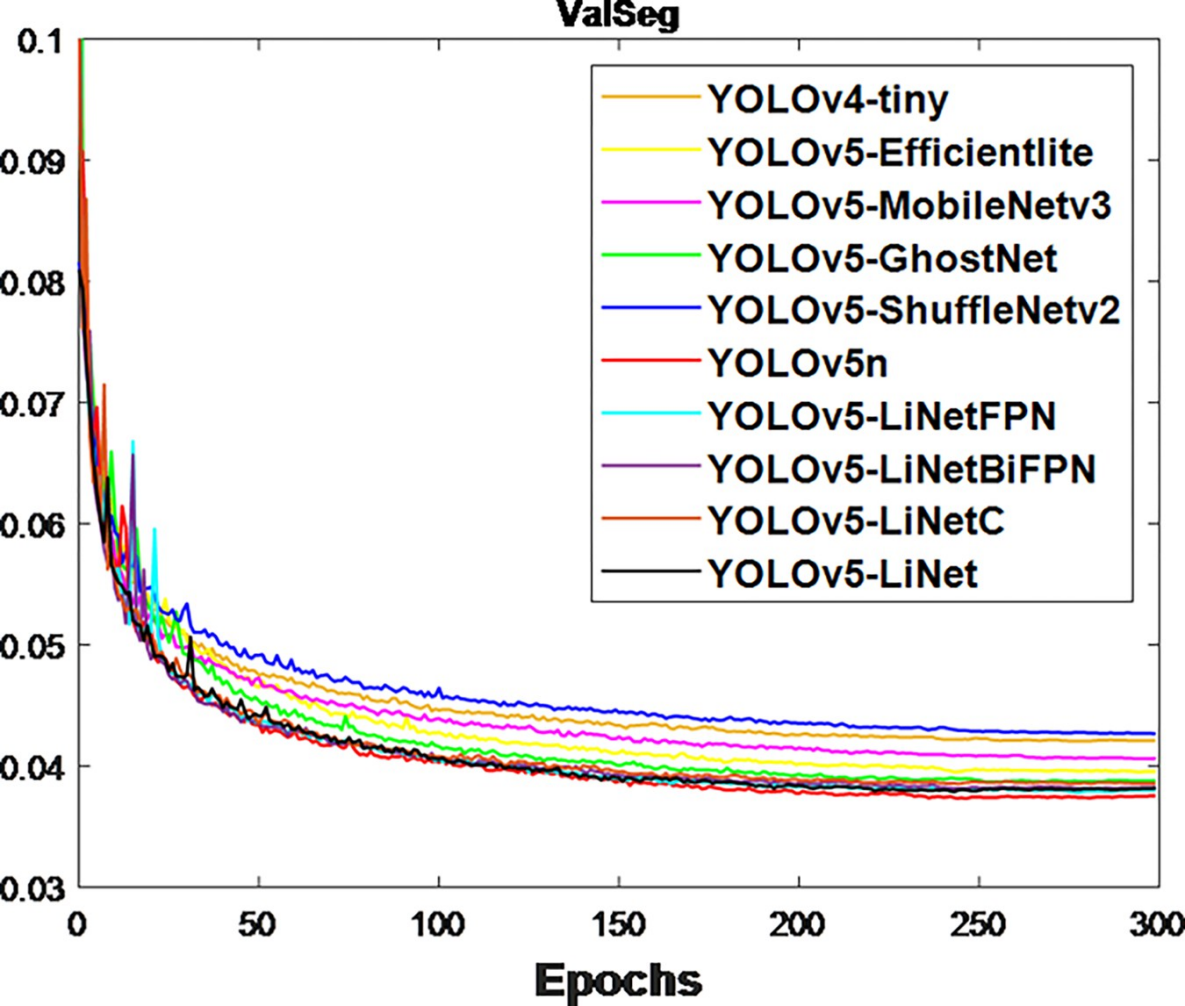
Output for segmentation validation loss.

**Fig 5:**
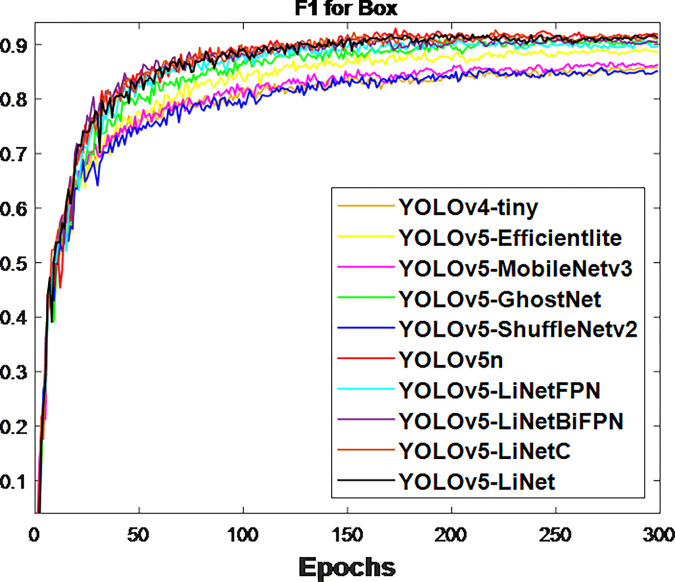
Output for box F_1_ score.

**Fig 6:**
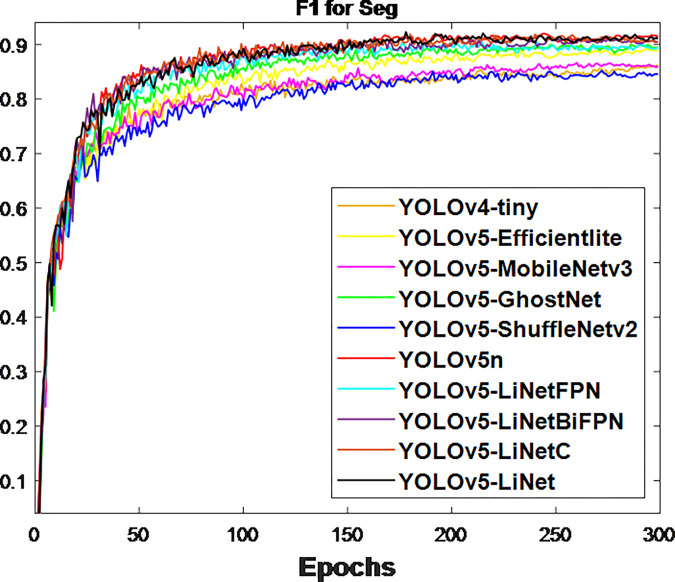
Output for segmentation F_1_ score.

**Fig 7:**
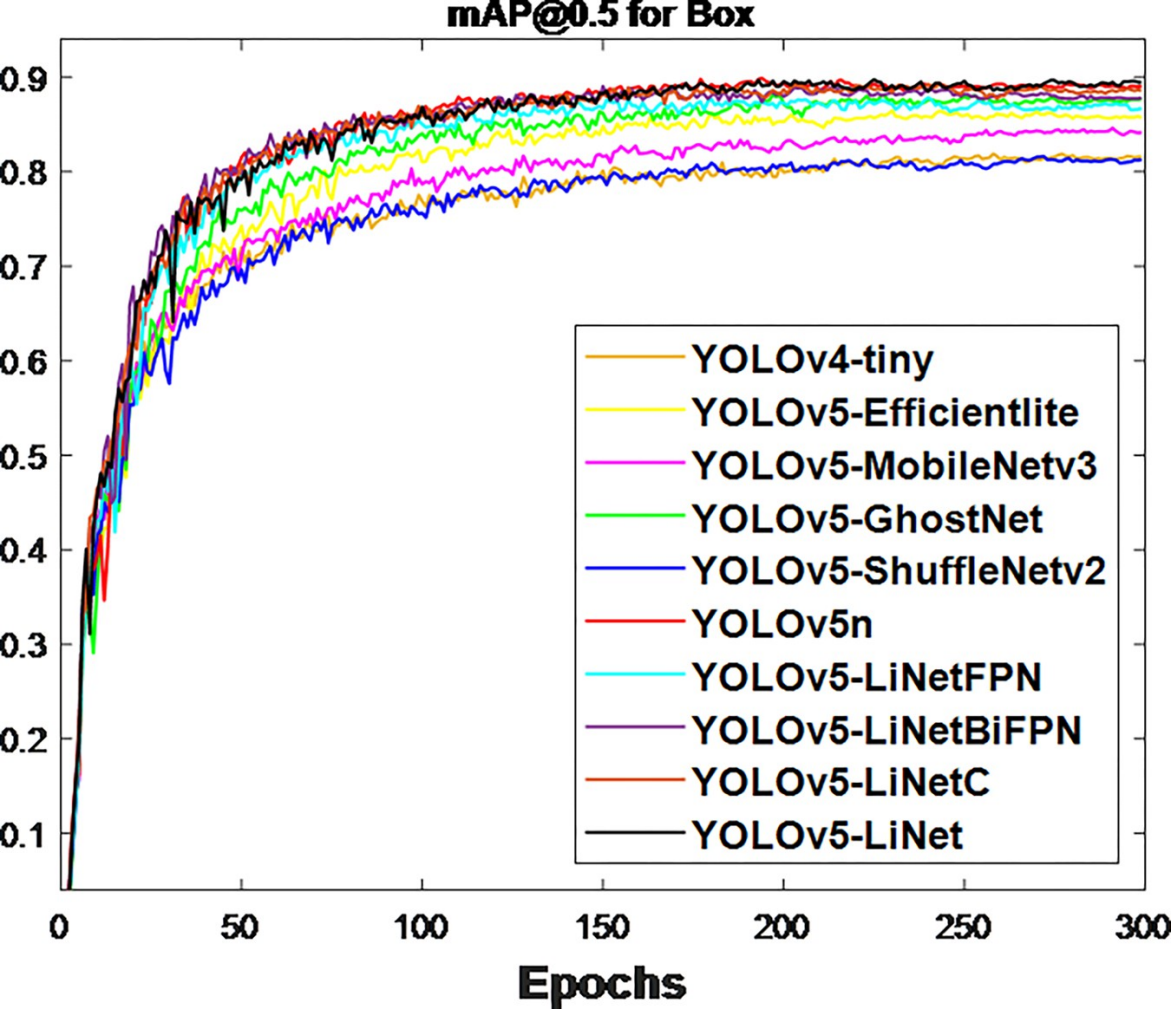
Output for box mAP@0.5.

**Fig 8:**
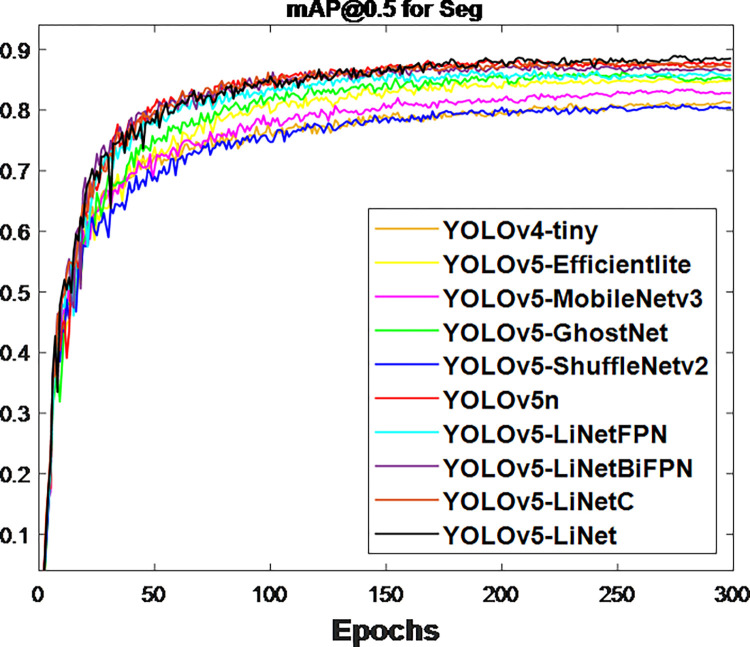
Output for segmentation mAP@0.5.

After the network training, the obtained validation loss for box and segmentation is presented in Figs 3 and 4 respectively. This is because validation loss measures how good the model fits valid set (new data) or predict. It was observed that the segmentation loss of all models in Fig 4 is lower than box loss in Fig 3, which is attributed to the bounding polygons that provides the actual shape of target. At the same time, the loss variations between models in Fig 4 is less than Fig 3. This is to justify the difference between using bounding boxes and polygons. The calculated F_1_-score for box and segmentation are displayed in Figs 5 and 6 respectively. Figs 5 and 6 shows that the F_1_-score of proposed YOLOv5-LiNet is more than other models, where YOLOv5-ShuffleNetv2 displayed the least F_1_-score. The mAP is more accurate than F_1_-score because it measures the global relationship between Precision and Recall. The depicted Figs 7 and 8 respectively show the mAP of box and segmentation. Just like F_1_-score, YOLOv5-LiNet outperformed other models in mAP. However, the displayed figures under F_1_-score and mAP for box is higher than that of segmentation. This is as a result of the complexity of polygon point of segmentation compared to rectangular point of the box.

**Fig 9:**
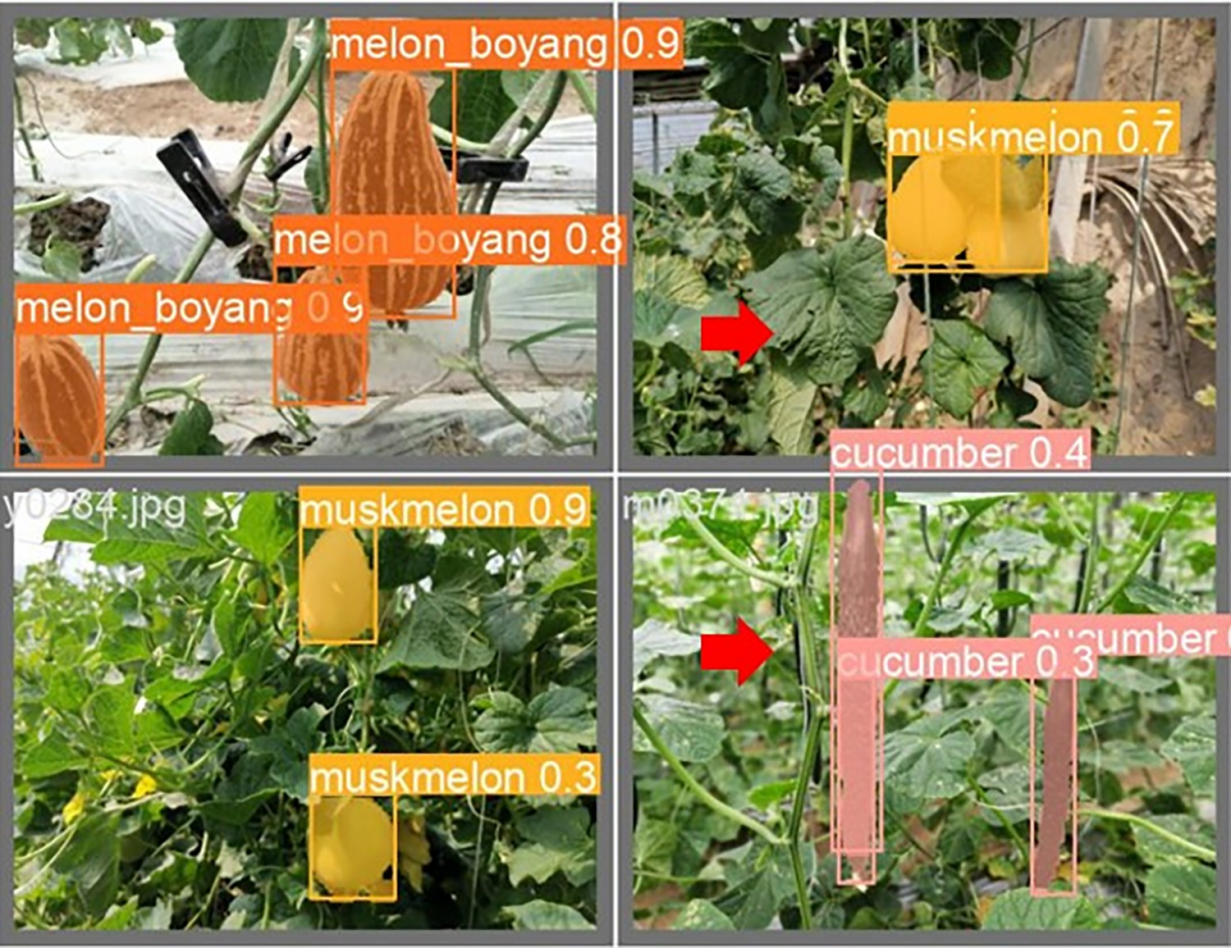
Image results of YOLOv4-tiny.

**Fig 10:**
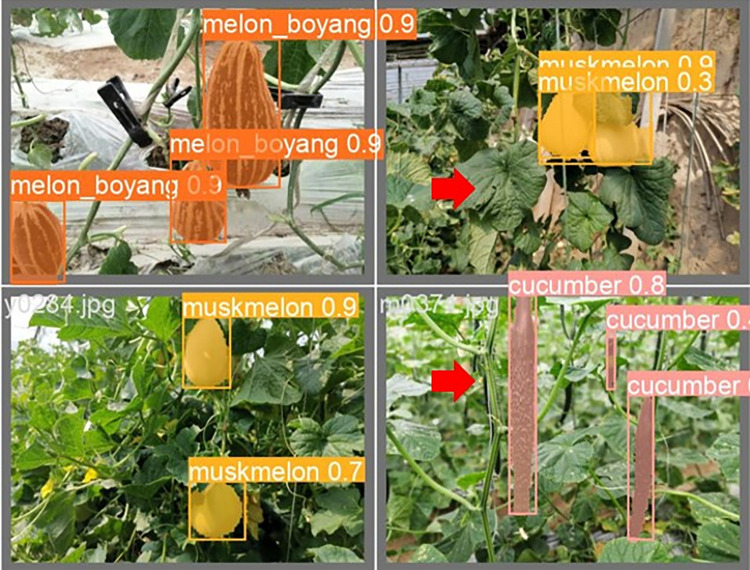
Image results of YOLOv5-Efficientlite.

**Fig 11:**
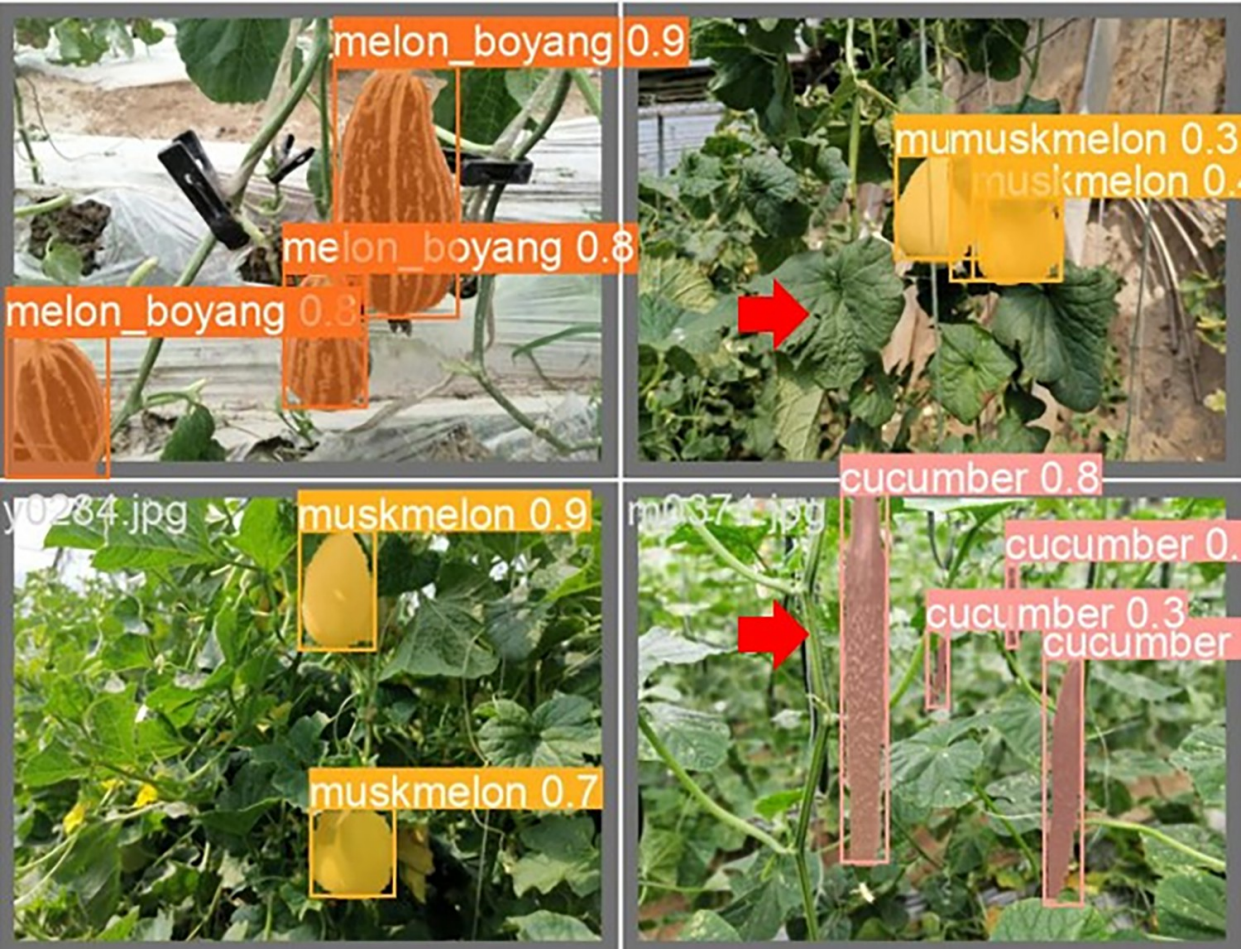
Image results of YOLOv5-MobileNetv3.

**Fig 12:**
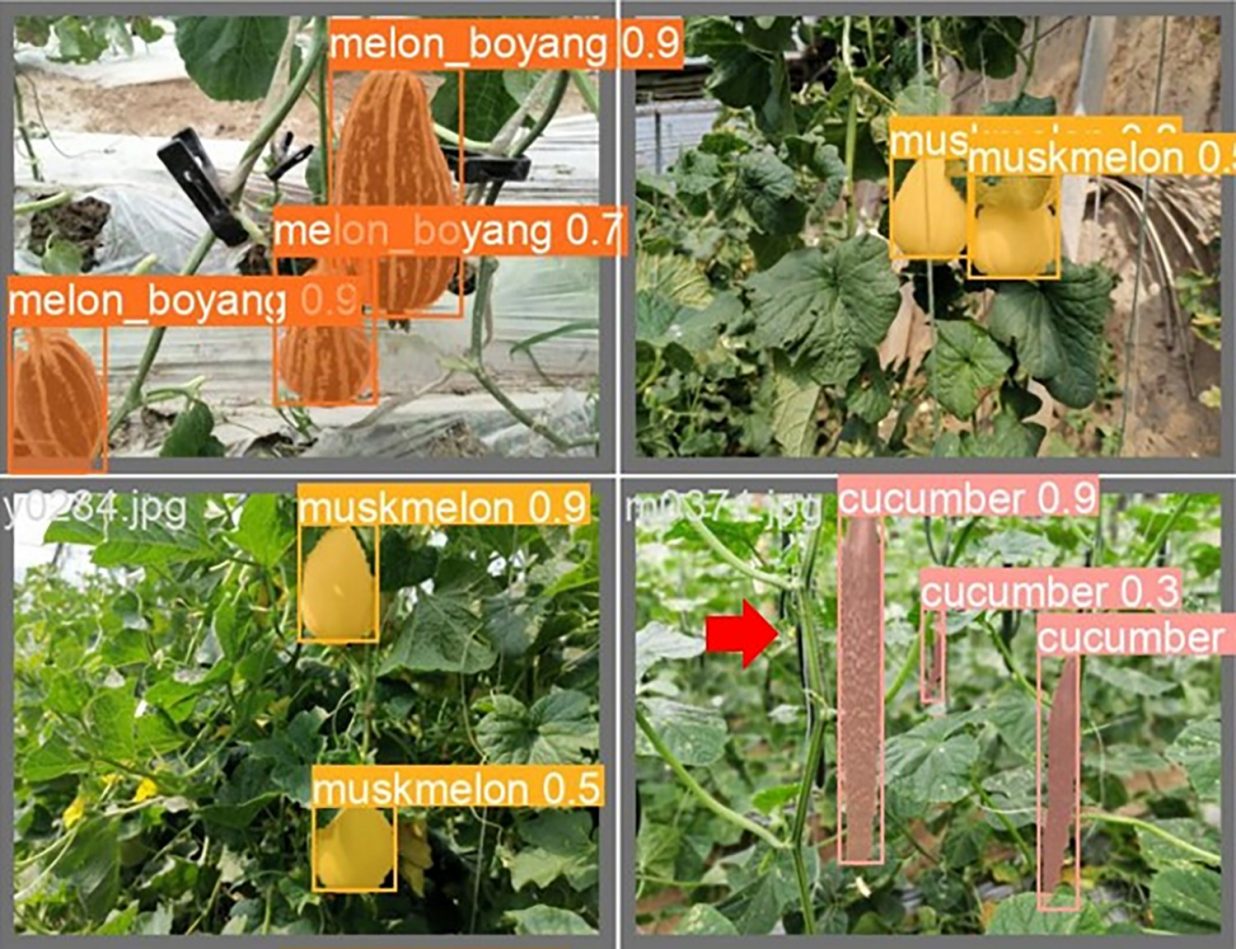
Image results of YOLOv5-GhostNet.

**Fig 13:**
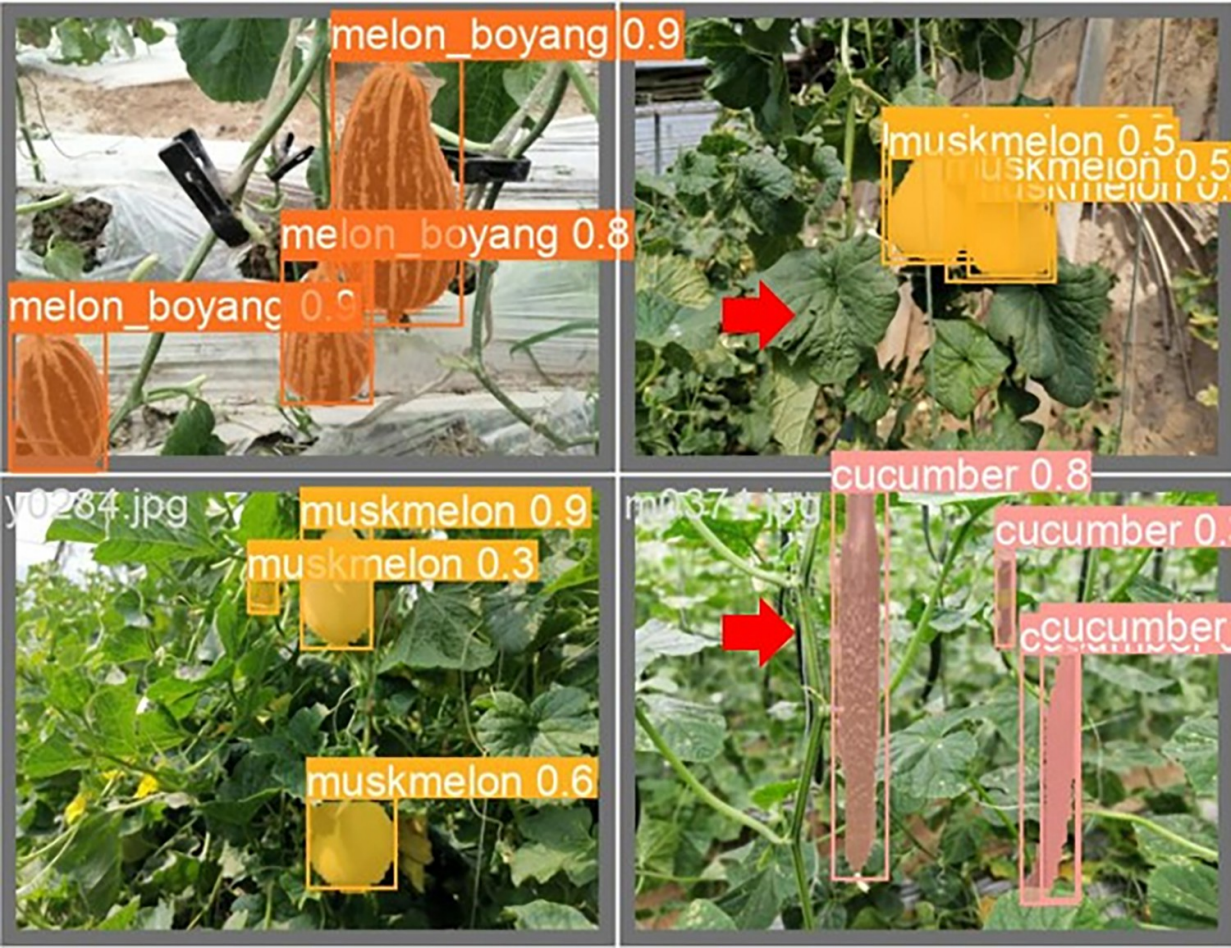
Image results of YOLOv5-ShuffleNetv2.

**Fig 14:**
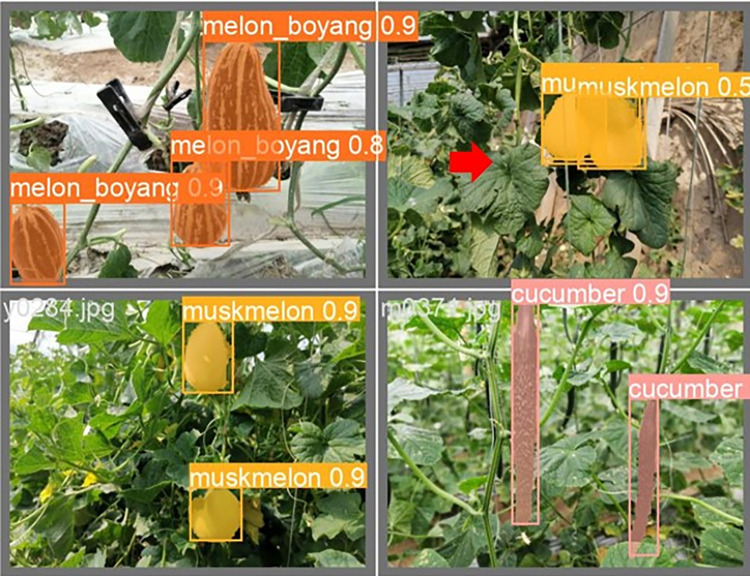
Image results of YOLOv5n.

**Fig 15:**
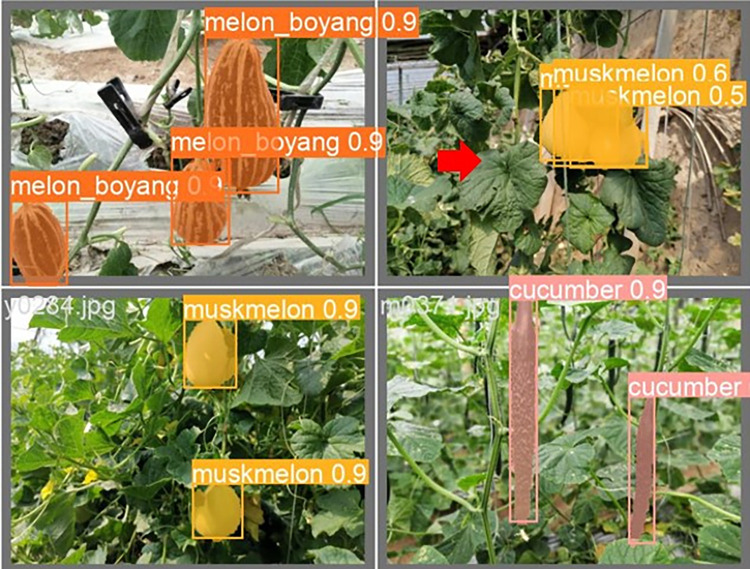
Image results of YOLOv5-LiNetFPN.

**Fig 16:**
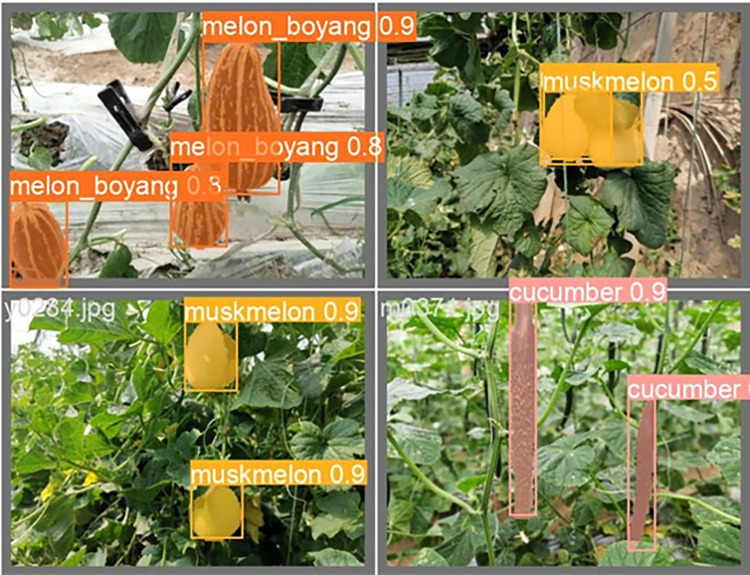
Image results of YOLOv5-LiNetBiFPN.

**Fig 17:**
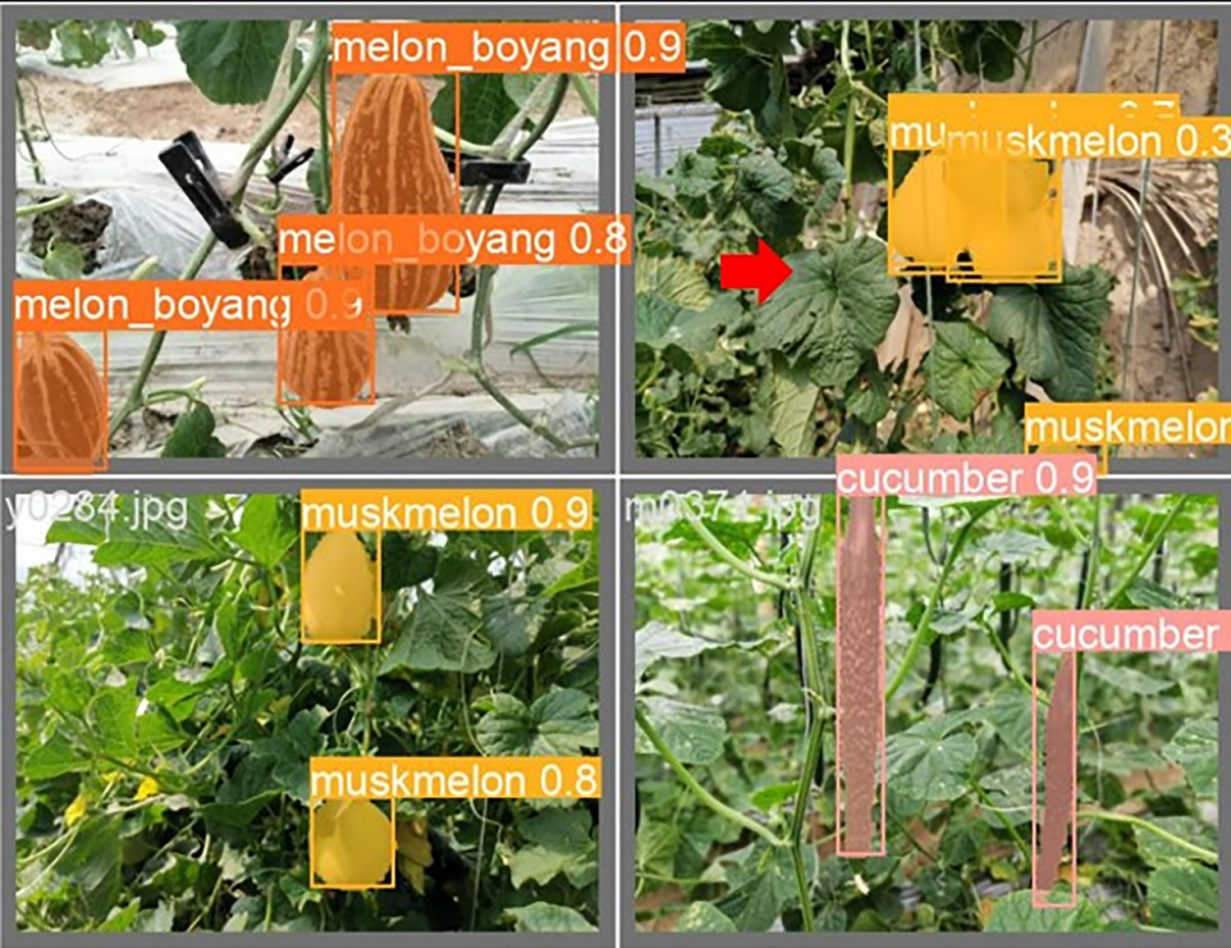
Image results of YOLOv5-LiNetC.

**Fig 18:**
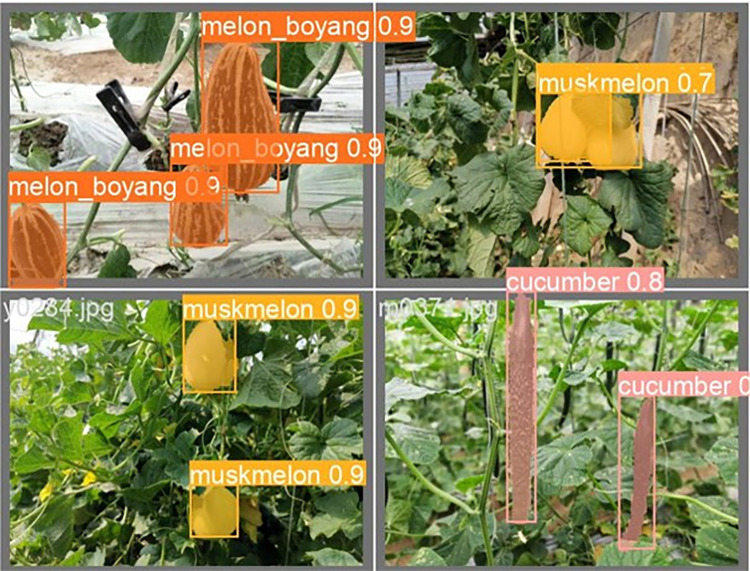
Image results of YOLOv5-LiNet.

The lightweight models were evaluated on test-set using four batch of images, and the obtained findings are shown in Figs 9‒18. A number of target fruits were detected and instantly segmented in the tested images without missed detection, showing robustness under various conditions. This is to prove the effectiveness of SPPF added into the models. Nevertheless, the level of detection accuracy varies from one to the other. Fig 9 of YOLOv4-tiny, Fig 10 of YOLOv5-Efficientlite, Fig 11 of YOLOv5-MobileNetv3 and Fig 13 of YOLOv5-ShuffleNetv2 shows two red arrow inaccurate detection in their images compared to Fig 12 of YOLOv5-GhostNet, Fig 14 of YOLOv5n, Fig 15 of YOLOv5-LiNetFPN and Fig 17 of YOLOv5-LiNetC with a single red arrow, while Fig 16 of YOLOv5-LiNetBiFPN and Fig 18 of YOLOv5-LiNet indicates accurate detection in their images. A prove to support the presented results in Figs 7 and 8 that YOLOv5-LiNet outperformed other models. Meanwhile, YOLOv5-LiNet trained with EIoU loss is more accurate compared to YOLOv5-LiNetC with applied CIoU loss function despite having the same network structure. This indicates that EIoU is better than CIoU as loss function, and requires more investigation.

The summary performance of tested compared models is shown in Table 2. Detection speed and accuracy are the main factors used to examine performance. Model weight and speed depends on layer for network topology, GFLOPs for speed of network, and size for weight of network, while accuracy is based on F_1_-score and mAP. Excluding the layer of Mask-RCNN, the obtained layer of YOLOv5-GhostNet is larger than other models, where YOLOv4-tiny is observed to have the least layer. The results of GFLOPs correspond to weight size derived through the parameter of a model. The obtained GFLOPs and weight size of Mask-RCNN is very large compared to YOLO-related models. This is to say that the lightweight size of a single-stage detector is far lesser than two-stage detector. Based on the YOLO-related models, the level of lightweight size is measure as YOLOv5n is greater than YOLOv5-GhostNet, YOLOv5-MobileNetv3, YOLOv5-LiNetBiFPN, YOLOv5-LiNetC, YOLOv5-LiNet, YOLOV5-LiNetFPN, YOLOv5-Efficientlite, YOLOv4-tiny and YOLOv5-ShuffleNetv2. This variation of weight size influences the tested real-time detection of model to support the claim of Lawal [8]. Apart from Mask-RCNN unable to meet the less than 50 ms standard of real-time detection proposed by Zhang et al. [40], all YOLO-related models were able to achieve this standard as shown in Table 2. YOLOv5-LiNet and YOLOv5-LiNetC having the same detection time of 2.6 ms is faster than 55.6 ms of Mask-RCNN, 2.8 ms of YOLOv5n, 3.4 ms of YOLOv5-Efficientlite, 3.5 ms of YOLOv5-MobileNetv3, 2.9 ms of YOLOv5-GhostNet, 2.9 ms of YOLOv5-LiNetBiFPN but slower than 2.4 ms of YOLOv5-LiNetFPN, 2.4 ms of YOLOv5-ShuffleNetv2 and 2.2 ms of YOLOv4-tiny. Nevertheless, the detection time of YOLOv5-LiNet is in close proximity with YOLOv5-LiNetFPN, YOLOv5-ShuffleNetv2 and YOLOv4-tiny. Adding detection accuracy to the resulting detection time serves to finalize the assessment of model performance. With reference to mAP, the stated results in Table 2 on accuracy is similar to the displayed results in Figs 7 and 8. Under mAP of box, 0.893 of YOLOv5-LiNet is 0.2%, 0.3%, 1.1%, 1.5%, 2.3%, 3.2%, 5.0%, 7.1%, 7.7% and 8.0% higher than YOLOv5-LiNetC, YOLOv5n, YOLOv5-LiNetBiFPN, YOLOv5-GhostNet, YOLOv5-LiNetFPN, YOLOv5-Efficientlite, YOLOv5-MoblieNetv3, Mask-RCNN, YOLOv4-tiny and YOLOv5-ShuffleNetv2 respectively. For mAP of instance segmentation, 0.885 of YOLOv5-LiNet is 0.5%, 1.0%, 1.3%, 2.2%, 2.6%, 3.3%, 5.6%, 5.8%, 7.2% and 7.5% more than YOLOv5-LiNetC, YOLOv5n, YOLOv5-LiNetBiFPN, YOLOv5-LiNetFPN, YOLOv5-GhostNet, YOLOv5-Efficientlite, YOLOv5-MoblieNetv3, Mask-RCNN, YOLOv4-tiny and YOLOv5-ShuffleNetv2 respectively. Owning to the outstanding performance of YOLOv5-LiNet compared to other models, the ablation study investigated using different neck network and loss function show that PANet > BiFPN > FPN and EIoU > CIoU respectively. The recorded mAP of instance segmentation on YOLOv5-LiNet increases by 0.5% using EIoU loss from YOLOv5-LiNetC, 1.3% using PANet from YOLOv5-LiNetBiFPN and 2.2% using PANet from YOLOv5-LiNetFPN. Additionally, YOLOv5-LiNet shows a better performance in terms of lightweight against proposed by Kang and Chen [17], Hurtik et al. [18] and Li et al. [19], accuracy and speed compared to state-of-art YOLOv5n and Mask-RCNN. For this reason, the YOLOv5-LiNet model is robust against the complex environment, accurate, fast, and applicable to low power computing devices embedded with computer vision.

**Table 2:** Compared summary performance of the tested models.

Models	Layers	GFLOPs	Size (MB)	F_1__B	F_1__S	mAP_B	mAP_S	t(ms)
Mask-RCNN	-	229.5	362.6	-	-	0.822	0.827	55.6
YOLOv4-tiny	139	4.3	2.1	0.857	0.861	0.816	0.813	2.2
YOLOv5-Efficientlite	225	4.8	2.6	0.890	0.886	0.861	0.852	3.4
YOLOv5-MobileNetv3	295	4.8	3.2	0.864	0.864	0.843	0.829	3.5
YOLOv5-GhostNet	317	5.5	3.2	0.903	0.900	0.878	0.859	2.9
YOLOv5-ShuffleNetv2	211	4.1	2.1	0.851	0.851	0.813	0.810	2.4
YOLOv5n	224	6.7	4.1	0.911	0.910	0.890	0.875	2.8
**Ablation study**								
YOLOv5-LiNetFPN	163	5.2	2.6	0.902	0.900	0.870	0.863	2.4
YOLOv5-LiNetBiFPN	182	5.6	3.2	0.906	0.902	0.882	0.872	2.9
YOLOv5-LiNetC	182	5.4	3.0	0.912	0.910	0.891	0.880	2.6
YOLOv5-LiNet	182	5.4	3.0	0.916	0.912	0.893	0.885	2.6

## 5. Conclusion

A lightweight YOLOv5-LiNet model for fruit instance segmentation has been suggested in this paper to consolidate fruit detection, based on the modified YOLOv5n for improved fruit production. The model comprised of Stem, Shuffle_Block, ResNet and SPPF as backbone network, PANet as neck network, and EIoU loss function to improve detection performance. At the same time, a robust cucurbit fruits image dataset with bounding polygon annotation was produced for comparative experiments on the proposed model. The ablation study carried out on YOLOv5-LiNet shows that the performance of applying PANet > BiFPN > FPN and EIoU > CIoU. YOLOv5-LiNet was compared with original YOLOv5n, YOLOv5-GhostNet, YOLOv5-MobileNetv3, YOLOv5-LiNetBiFPN, YOLOv5-LiNetC, YOLOv5-LiNet, YOLOv5-LiNetFPN, YOLOv5-Efficientlite, YOLOv4-tiny and YOLOv5-ShuffleNetv2 of lightweight model including Mask-RCNN. The obtained results demonstrated that YOLOv5-LiNet with 0.893 of mAP box, 0.885 of mAP instance segmentation mAP, 3.0 MB of weight size and 2.6 ms of detection time combined together is outstanding in performance compared to other lightweight models. Hence, the YOLOv5-LiNet model is highly robust against complex and changeable environment, accurate, prospective for better generalization and real-time detection, applicable to low power computing devices and extendable to other agricultural products for instance segmentation.

## Supporting information

S1 Data (RAR)

## References

[1] Shahbandeh M. Global fruit production in 2020. 2022; https://www.statista.com/statistics/264001/worldwide-production-of-fruit-by-variety/

[2] Rocha R, Hauagge DC, Wainer J, Goldenstein S. Automatic fruit and vegetable classification from images. Comput. Electron. Agric. 2010; 70: 96–104. 10.1016/j.compag.2009.09.002

[3] Sharpe SM, Schumann AW, Boyd NS. Goosegrass detection in strawberry and tomato using a convolutional neural network. Sci. Rep. 2020; 10: 9548. doi: 10.1038/s41598-020-66505-9 32533076 PMC7293330

[4] Lawal MO. Tomato detection based on modified YOLOv3 framework. Sci Rep. 2021a; 11: 1447 doi: 10.1038/s41598-021-81216-5 33446897 PMC7809275

[5] He K, Gkioxari G, Dollár P, Girshick R. Mask-RCNN. Proc. IEEE Int. Conf. Comput. Vis. 2017; 2961–2969. 10.1109/ICCV.2017.322

[6] Koirala A, Walsh KB, Wang Z, McCarthy C. Deep learning for real time fruit detection and orchard fruit load estimation: benchmarking of ‘MangoYOLO’. Precision Agriculture. 2019; 20: 1107−1135.

[7] Koirala A, Walsh KB, Wang Z, McCarthy C. Deep learning–Method overview and review of use for fruit detection and yield estimation. Comput. Electron. Agric. 2019; 162: 219−234.

[8] Lawal MO. YOLOMuskmelon: Quest for Fruit Detection Speed and Accuracy Using Deep Learning. IEEE Access. 2021b; 9: 15221−15227.

[9] Lawal MO. Development of tomato detection model for robotic platform using deep learning. Multimed Tools Appl. 2021c; 80: 26751–26772. 10.1007/s11042-021-10933-w

[10] Shang Y, Wang Y, Liu B. RiceNet: a lightweight instance segmentation network for adhesive rice grains. International Conference on Wireless Communications and Smart Grid (ICWCSG). 2021; p. 258–261, 10.1109/ICWCSG53609.2021.00056

[11] Liu X, Zhao D, Jia W, Ji W, Ruan C, Sun Y, et al. Cucumber fruits detection in greenhouses based on instance segmentation. IEEE Access. 2019; 7: 139635–139642. 10.1109/ACCESS.2019.2942144

[12] Yu Y, Zhang K, Yang L, Zhang D. Fruit detection for strawberry harvesting robot in non-structural environment based on mask-RCNN. Comput. Electron. Agricult. 2019; 163.

[13] Ilyas T, Khan A, Umraiz M, Jeong Y, Kim H. Multi-scale context aggregation for strawberry fruit recognition and disease phenotyping. IEEE Access. 2021; 9: 124491–124504. 10.1109/ACCESS.2021.3110978

[14] Jia W, Wei J, Zhang Q, Pan N, Niu Y, Yin X, et al. Accurate segmentation of green fruit based on optimized mask RCNN application in complex orchard. Front. Plant Sci. 2022; 13: 955256. doi: 10.3389/fpls.2022.955256 36035694 PMC9399748

[15] Howard A, Sandler M, Chen B, et al. Searching for MobileNetV3, IEEE/CVF International Conference on Computer Vision (ICCV), Seoul, South Korea. 2019; p. 1314–1324, 10.1109/ICCV.2019.00140

[16] Hameed Khurram, Chai Douglas, Rassau Alexander. Score-based mask edge improvement of Mask-RCNN for segmentation of fruit and vegetables. Expert Systems with Applications. 2021; 190: 116205. 10.1016/j.eswa.2021.116205

[17] Kang H, Chen C. Fruit detection, segmentation and 3D visualization of environments in apple orchards. Comput. Electron. Agricult. 2020; 171: 105302. 10.1016/j.compag.2020.105302

[18] Hurtik Petr, Molek Vojtech, Hula Jan, Vajgl Marek, Vlasanek Pavel, and Nejezchleba Tomas. Poly-yolo: higher speed, more precise detection and instance segmentation for yolov3. 2020; arXiv preprint, arXiv: 2005.13243

[19] Li Fengze, Ma Jieming, Tian Zhongbei, Ge Ji, Liang Hai-Ning, Zhang Yungang, et al. Mirror-YOLO: An Attention-Based Instance Segmentation and Detection Model for Mirrors. 2022; 10.48550/arXiv.2202.08498

[20] Jocher G, Stoken A, Borovec J, et al. ultralytics/yolov5: v7.0 (Version v3.0). 2022; Zenodo

[21] Howard AG, Zhu M, Chen B, Kalenichenko D, Wang W, Weyand T, et al. MobileNets: efficient Convolutional Neural Networks for Mobile Vision Applications. 2017; arXiv preprint, arXiv: 1704.04861.

[22] Sandler M, Howard A, Zhu M, Zhmoginov A, Chen L. MobileNetV2: Inverted Residuals and Linear Bottlenecks. In Proceedings of the 2018 IEEE Conference on Computer Vision and Pattern Recognition (CVPR), Salt Lake City, UT, USA. 2018: p. 4510–4520.

[23] Landola FN, Han S, Moskewicz MW, et al. Squeezenet: Alexnet-level accuracy with 50x fewer parameters and< 0.5 MB model size. 2016; arXiv preprint, arXiv:1602.07360.

[24] Gholami A, Kwon K, Wu B, et al. SqueezeNext: Hardware-Aware Neural Network Design IEEE/CVF Conference on Computer Vision and Pattern Recognition Workshops (CVPRW). Salt Lake City, UT. 2018; p. 1719–1728

[25] Zhang X, Zhou X, Lin M, Sun J. ShuffleNet: An Extremely Efficient Convolutional Neural Network for Mobile Devices. Computer Vision and Pattern Recognition. 2017; arXiv preprint, arXiv: 1707.01083v2 10.48550/arXiv.1707.01083

[26] Ma N, Zhang X, Zheng HT, et al. ShuffleNetV2: Practical Guidelines for Efficient CNN Architecture Design. European Conference on Computer Vision. Springer, Cham. 2018; p. 122–138

[27] Adarsh P, Rathi P, Kumar M. YOLOv3-Tiny: Object Detection and Recognition using one stage improved model. 2020 6th International Conference on Advanced Computing and Communication Systems (ICACCS), Coimbatore, India. 2020; p. 687–694

[28] Bochkovskiy A, Wang CY, Liao HYM. YOLOv4: Optimal speed and accuracy of object detection. 2020; arXiv preprint, arXiv: 2004.10934v1.

[29] Chen M, Tang Y, Zou X, Huang Z, Zhou H, Chen S, et al. 3D global mapping of large-scale unstructured orchard integrating eye-in-hand stereo vision and SLAM. Comput. Electron. Agricult. 2021; 187: 106237. 10.1016/j.compag.2021.106237

[30] Wei J, Ding Y, Liu J, Ullah MZ, Yin X, Jia W, et al. Novel green-fruit detection algorithm based on D2D framework. J. Int. J. Agricult. Biol. Eng. 2022; 15: 251–259. 10.25165/j.ijabe.20221501.6943

[31] Wada K. v5.0.5. 2020; https://github.com/wkentaro/labelme

[32] Wang Robert J., Li Xiang, Ling Charles X. Pelee: A real-time object detection system on mobile devices. NeurIPS. 2018; arXiv preprint, arXiv: 1804.06882

[33] He K, Zhang X, Ren S, Sun J. Deep residual learning for image recognition. IEEE Conference on Computer Vision and Pattern Recognition (CVPR), Las Vegas. 2016; p. 770–778

[34] Stefan E, Eiji U, Kenji D. Sigmoid-weighted linear units for neural network function approximation in reinforcement learning. 2017; arXiv preprint, arXiv: 1702.03118. 10.1016/j.neunet.2017.12.012 29395652

[35] He K, Zhang X, Ren S, Sun J. Spatial pyramid pooling in deep convolutional networks for visual recognition. IEEE Transactions on Pattern Analysis and Machine Intelligence (TPAMI). 2015; 37, p. 1904–1916 doi: 10.1109/TPAMI.2015.2389824 26353135

[36] Liu S, Qi L, Qin H, Shi J, Jia J. Path aggregation network for instance segmentation. In Proceedings of the IEEE Conference on Computer Vision and Pattern Recognition (CVPR). 2018; p. 8759–8768.

[37] Zhang Y, Ren W, Zhang Z, Jia Z, Wang L, Tana T. Focal and Efficient IOU Loss for Accurate Bounding Box Regression. 2022; arXiv: 2101.08158v2 https://arxiv.org/pdf/2101.08158.pdf

[38] Zheng Z, Wang P, Liu W, Li J, Ye R, Ren D. Distance-IoU Loss: Faster and better learning for bounding box regression. 2019; arXiv preprint, arXiv: 1911.08287v1

[39] iscyy. YOLOAir: Makes improvements easy again. 2022; https://github.com/iscyy/yoloair

[40] Zhang W, Liu Y, Chen K, Li H, Duan Y, Wu W, et al. Lightweight Fruit-Detection Algorithm for Edge Computing Applications. Front. Plant Sci. 2021; 12: 740936. doi: 10.3389/fpls.2021.740936 34721466 PMC8548576

